# Strain-induced switching of heat current direction generated by magneto-thermoelectric effects

**DOI:** 10.1038/s41598-019-49567-2

**Published:** 2019-09-13

**Authors:** Shinya Ota, Ken-ichi Uchida, Ryo Iguchi, Pham Van Thach, Hiroyuki Awano, Daichi Chiba

**Affiliations:** 1 Department of Applied Physics, The University of Tokyo, Bunkyo, Tokyo 113-8656 Japan; 2 Institute of Scientific and Industrial Research, Osaka University, Ibaraki, Osaka 567-0047 Japan; 3 National Institute for Materials Science, Tsukuba, 305-0047 Japan; 4 Center for Spintronics Research Network, Tohoku University, Sendai, 980-8577 Japan; 5 Department of Mechanical Engineering, The University of Tokyo, Bunkyo, Tokyo 113-8656 Japan; 6 Toyota Technological Institute, Nagoya, 468-8511 Japan; 7 Institute of Materials Science, Vietnam Academy of Science and Technology, 18-Hoang Quoc Viet, Hanoi, Vietnam; 8 Center for Spintronics Research Network at Osaka University, Toyonaka, Osaka 560-6671 Japan

**Keywords:** Spintronics, Applied physics

## Abstract

Since the charge current plays a major role in information processing and Joule heating is inevitable in electronic devices, thermal management, i.e., designing heat flows, is required. Here, we report that strain application can change a direction of a heat current generated by magneto-thermoelectric effects. For demonstration, we used metallic magnets in a thin-film form, wherein the anomalous Ettingshausen effect mainly determines the direction of the heat flow. Strain application can alter the magnetization direction owing to the magnetoelastic effect. As a result, the heat current, which is in the direction of the cross product of the charge current and the magnetization vector, can be switched or rotated simply by applying a tensile strain to the metallic magnets. We demonstrate 180° switching and 90° rotation of the heat currents in an in-plane magnetized Ni sample on a rigid sapphire substrate and a perpendicularly magnetized TbFeCo film on a flexible substrate, respectively. An active thermography technique was used to capture the strain-induced change in the heat current direction. The method presented here provides a novel method for controlling thermal energy in electronic devices.

## Introduction

Thermal management is becoming more and more important with the greater miniaturization and performance enhancement of electronic devices. Spin caloritronics^1^, which is a field triggered by observation of the spin Seebeck effect^2^, may help control heat by using spin or magnetization in materials. Among a variety of spin-caloritronic and magneto-thermoelectric phenomena, the spin Peltier effect (SPE)^3–6^ and the anomalous Ettingshausen effect (AEE)^7^ are candidates for active thermal management. One of their novel points is the controllability of a heat current ***J***_q_ with a magnetic field or a magnetization ***M***. In the AEE, ***J***_q_, generated by the charge current ***J***_c_, follows the relation ***J***_q_ ∝ ***J***_c_ × ***M***^7^, indicating that the ***J***_q_ direction can be rotated or reversed by changing the ***M*** direction. Although the AEE has been known for a long time, such demonstrations and detailed studies have recently been realized by using a lock-in thermography (LIT) technique^4,5,7–13^.

**Figure 1:**
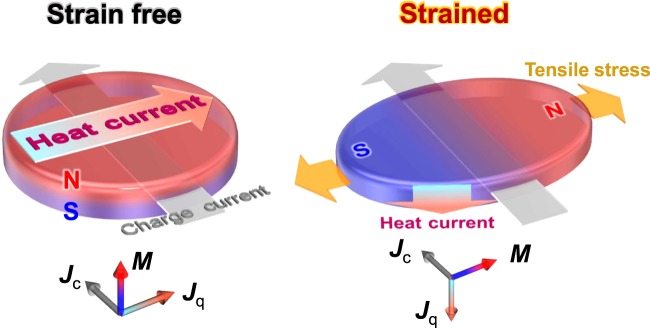
Schematic illustration of strain-induced switching of a heat flow. The AEE induced by a charge current ***J***_c_ in magnetic materials creates a resultant heat flow ***J***_q_, which is in the direction of the cross product of ***J***_c_ and the magnetization ***M*** (see the vector relation for ***J***_c_, ***J***_q_, and ***M*** shown below the schematics). The direction of ***J***_q_ can be switched by stress application because an additional introduced strain can alter the magnetic easy axis (the direction of ***M***) through the magnetoelastic effect. The schematic here shows the 90° switching of ***J***_q_ induced by the strain-induced rotation of ***M*** from the perpendicular (strain-free) to the in-plane direction (strained).

In this paper, we report that mechanically induced strain can switch the direction of the heat currents generated by the magneto-thermoelectric effects through the magnetoelastic coupling. The magnetoelastic effect enables control of the ***M*** direction by using strain instead of using a magnetic field when the strain and/or the magnetostriction constant of the magnetic material are large enough. Previously, a strain of more than 1% was applied to magnetic thin films fabricated on flexible substrates, which resulted in reversible magnetic easy axis switching^14,15^. To demonstrate the strain-induced switching of ***J***_q_ induced by the AEE, an in-plane magnetized ferromagnetic Ni film and a perpendicularly magnetized ferrimagnetic TbFeCo thin film deposited respectively on sapphire and flexible polyethylene-naphthalate (PEN) substrates were prepared. In the Ni sample, a 180° reversal of ***J***_q_ was observed at an in-plane bias field by bending the sample. In the TbFeCo sample, stretching the PEN substrate could switch the magnetic easy axis from the perpendicular-to-plane to the in-plane direction^14^, resulting in a 90° rotation of ***J***_q_ at zero external magnetic field, as schematically depicted in Fig. 1. This heat-control function may offer an unconventional approach for thermal management technologies.

**Figure 2:**
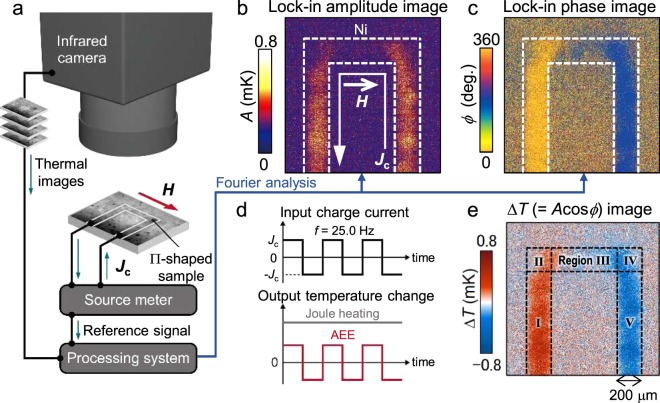
Lock-in thermography measurements. (**a**) A schematic illustration of the LIT system. The temperature modulation synchronized with the output of a source metre is extracted by Fourier analysis through a processing system. (**b**,**c**) Images of a lock-in amplitude *A* and phase *ϕ*, respectively. The Π-shaped Ni wire resides in the area surrounded by the dashed lines. (**d**) Time charts of the input charge current applied to the sample and the expected output, *i*.*e*., time-dependent temperature changes induced by the AEE and Joule heating. (**e**) Δ*T* (=*A*cos*ϕ*) image calculated from b and c. The thermal images shown here were taken using the Ni sample at *μ*_0_*H*_*y*_ = +5.6 mT without bending the substrate. Regions I-V are defined by the black dashed lines in (**e**). To enhance the infrared emissivity and ensure uniform emission properties, the surfaces of the samples were coated with insulating black ink, of which the emissivity is >95%.

Temperature modulation due to the AEE-induced ***J***_q_ in the samples was detected using the LIT technique at room temperature, as schematically explained in the panels of Fig. 2. For the present experiments, the Ni and TbFeCo films on the substrates were processed into 200-μm-wide Π-shaped wires. An alternating charge current with a rectangular waveform, zero offset, and a frequency *f* of 25 Hz was applied to the wire using a current source metre. Although the Joule heating generated by such an alternating current is constant over time, the temperature modulation due to the AEE oscillates with *f*, because the AEE is linearly responsive to the charge current [see Fig. 2d]. Thus, the temperature increase due to Joule heating can be eliminated by extracting the linear response contribution of the temperature modulation via Fourier analysis, enabling pure detection of the AEE. By this LIT measurement, the spatial distributions of the amplitude *A* and phase *ϕ* of the AEE-induced temperature modulation are obtained, where *ϕ* represents the sign of the temperature modulation depending on the charge current with the time delay due to thermal diffusion. Typical examples of the *A* and *ϕ* images for the Ni sample are shown in Fig. 2b,c, respectively. Figure 2e shows the resultant Δ*T* (=*A*cos*ϕ*) image, where Δ*T* is the current-induced temperature modulation with the sign information when the time delay due to thermal diffusion is negligibly small^11^, and this condition can be applied to our experiments.

## Results

### Strain-induced modulation of heat current switching field

**Figure 3:**
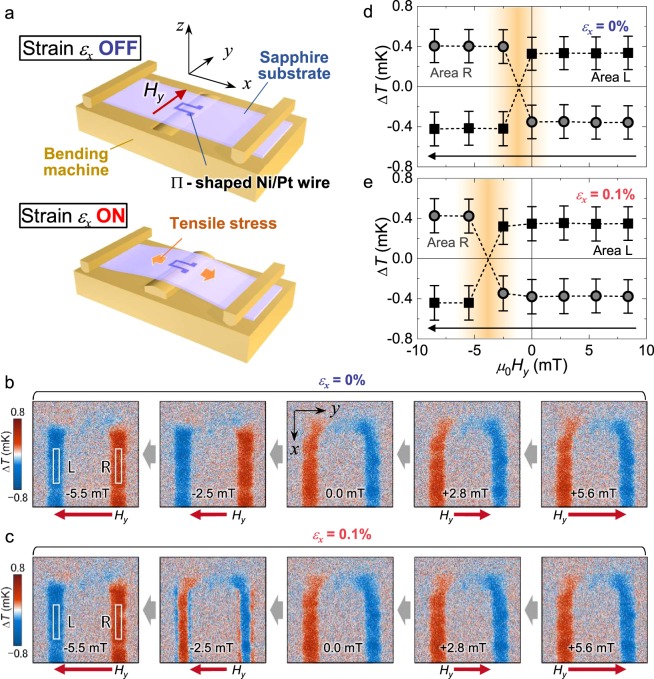
Strain-induced modulation of a heat current switching field using the Ni sample. (**a**) A schematic illustration of the experimental setup. *ε*_*x*_ was introduced to the Ni film using a three-point bending machine. The electrical connections are omitted from the illustration for simplicity. (**b**,**c**) Δ*T* images without bending (*ε*_*x*_ = 0%) and with bending (*ε*_*x*_ = 0.1%), respectively. In both measurements, *μ*_0_*H*_*y*_ was reduced from +5.6 mT to -5.5 mT, and the images were taken using the LIT technique at each *H*_*y*_. (**d**,**e**) Δ*T* as a function of *μ*_0_*H*_*x*_ for the cases of *ε*_*x*_ = 0% and 0.1%, respectively. The Δ*T* plotted here is the average of the temperature modulation over areas L (square data plots) and R (circle data plots) indicated in b and c. The error bar corresponds to the standard deviation. Although 180° switching of ***J***_q_ along the *z* axis was observed in both cases, the switching field was different between the two because of the strain-induced enhancement of the coercivity.

We firstly show the experimental results of the strain-induced modulation of the 180° switching of the ***J***_q_ direction using the in-plane-magnetized Ni sample. Thermal images of this sample were obtained by injecting the alternating charge current with an amplitude *J*_c_ of 10 mA under an applied in-plane magnetic field along the *y* axis (*H*_*y*_). A tensile strain *ε*_*x*_ was introduced to the Ni film along the *x* axis by bending the substrate using a three-point bending machine made of brass [see Fig. 3a and Methods]. The strain applied to the sample surface was calculated using the displacement of the sample centre and the thickness of the substrate. Ni has a negative magnetoelastic constant^15^, *i*.*e*., Ni becomes easier to magnetize along the *y* axis when the substrate is bent in the present configuration. We note that the initial magnetic anisotropy of regions I and V [see Fig. 2e for a definition of the regions] is along the *y* axis because a separate measurement confirms that a *μ*_0_*H*_*x*_ of ~10 mT is needed at *ε*_*x*_ = 0% to completely saturate ***M*** in these regions toward the *x* direction. A tiny built-in strain potentially introduced during sputter-deposition might be attributed to the initial magnetic anisotropy of the sample. In the following discussion, we focus on regions I and V because in the present configuration, the AEE-originated temperature modulation is expected to be seen in these regions, where ***J***_c_ (***M***) is along the *x* (*y*) direction^7^.

Figure 3b,c) shows the Δ*T* images for *ε*_*x*_ = 0 and 0.1%. The five images shown as a series of panels were taken by changing *H*_*y*_ from positive to negative. At any *H*_*y*_ value, the surface temperature in regions I and V is clearly increased or decreased, indicating that ***J***_q_ is along the *z* axis. The sign of Δ*T* in these regions was observed to be reversed by reversing the direction of ***J***_c_. This is consistent with the symmetry of the AEE (see Fig. 1). In the negative *H*_*y*_ region, the sign reversal of Δ*T* occurs in both *ε*_*x*_ = 0 and 0.1% cases. The reversal is observed in the image at *μ*_0_*H*_*y*_ = −2.5 mT for *ε*_*x*_ = 0%, indicating that the coercivity is below 2.5 mT. For the case of *ε*_*x*_ = 0.1%, however, *μ*_0_*H*_*y*_ = −5.5 mT is needed for completely switching the sign of Δ*T*. This difference is attributed to the strain-induced enhancement of the coercivity. The direction of ***J***_q_ between *ε*_*x*_ = 0 and 0.1% is opposite at *μ*_0_*H*_*y*_ = −2.5 mT, showing that the strain can reverse the ***J***_q_ direction by 180°. Note that ***M*** reversal only at the edge is seen at *μ*_0_*H*_*y*_ = −2.5 mT and *ε*_*x*_ = 0.1%. This is most likely due to the weaker magnetic anisotropy because of the smaller thickness at the edge. Figure 3d,e summarises the behaviour of Δ*T* as a function of *μ*_0_*H*_*y*_ for the *ε*_*x*_ = 0 and 0.1% cases, respectively. In the figures, the averaged Δ*T* values over areas L and R indicated in Fig. 3b,c are plotted, which clearly shows the strain-induced enhancement of the switching field. In region III, ***J***_c_ and ***M*** are expected to be parallel to each other if the magnetic anisotropy is uniform along the whole wire. In fact, the magnitude of Δ*T* for region III is observed to be much smaller than that for regions I and V, consistent with the symmetry of the AEE [see Fig. 3b,c].

### Strain-induced 90° rotation of heat current without an external magnetic field

**Figure 4:**
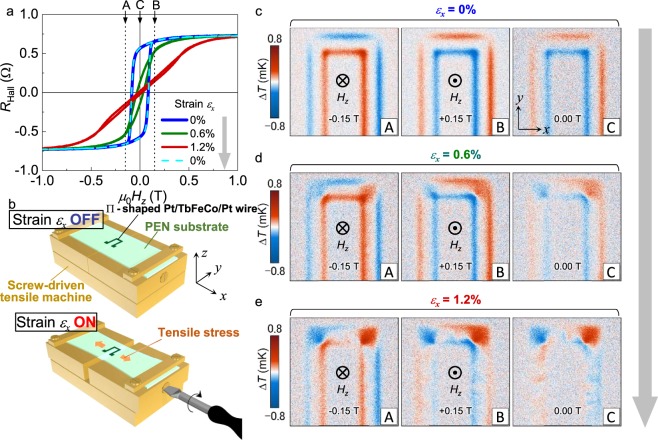
Strain-induced 90° switching of a heat current using the TbFeCo sample. (**a**) The results of the magnetic hysteresis measurement using the anomalous Hall effect. *H*_*z*_ was swept, and *R*_Hall_ was measured under different in-plane tensile strain conditions. (**b**) A schematic illustration of the experimental setup for the LIT measurement. *ε*_x_ was introduced by stretching the substrate. The electrical connections are omitted from the illustration for simplicity. (**c**–**e**) Δ*T* images taken under three different *H*_*z*_ conditions [see dashed lines A-C in a] for *ε*_x_ = 0, 0.6, and 1.2%, respectively. Here, the magnetic field-independent background signals were subtracted from the raw LIT images, where the background was calculated from the raw images at *μ*_0_*H*_*z*_ = ±0.15 T and *ε*_*x*_ = 0%. Complete elimination of the in-plane heat current is realised at *ε*_x_ = 1.2% and *μ*_0_*H*_*z*_ = 0 T because of the easy axis switching of ***M*** from the perpendicular to in-plane direction.

Secondly, the 90° switching of ***J***_q_ is demonstrated using the perpendicularly magnetized TbFeCo sample. In this experiment, a screw-driven tensile machine made of brass was used to stretch the PEN substrate [Fig. 4b]. In this case, the value of the applied strain *ε*_*x*_ was calibrated using the elongation of the sample measured via a microscope and the rotation number of the screw.

Figure 4a shows the results of hysteresis measurements under different strain conditions. The Hall resistance *R*_Hall_, which is proportional to the *z*-component of ***M*** because of the dominant contribution from the anomalous Hall effect, was measured by sweeping the external magnetic field along the *z* direction (*H*_*z*_). A Hall bar-shaped TbFeCo formed on the PEN substrate was used in this experiment (see Methods). As in the case of our previous report^14^, clear easy axis switching from the perpendicular-to-plane direction to the in-plane direction was observed by increasing the strain. A separate experiment confirms that the magnetization easy axis under the strain application is along the *x* direction, which is consistent with the positive magnetoelastic constant of TbFeCo^14,16^. By returning the strain to zero, the shape of the hysteresis loop reproducibly returned to a similar shape as that of the initial loop. We note that the remanent magnetization of *ε*_*x*_ = 0% is smaller than the saturation magnetization, which is most likely attributed to the reduced magnetization due to randomly canted spin structures^17^.

Next, the results of the LIT measurement using the Π-shaped TbFeCo wire are discussed. In the configuration employed for the present experiment [Fig. 4b], regions I and V are along the *y* axis. The Δ*T* images obtained at *μ*_0_*H*_*z*_ = (A) −0.15 T, (B) +0.15 T, and (C) 0 T for *ε*_*x*_ = 0, 0.6, and 1.2% are respectively shown in Fig. 4c–e. The LIT images of this sample were obtained by injecting the alternating charge current with *J*_c_ = 5 mA. *H*_*z*_ was changed in the order of A → B → C. Here, the magnetic field-independent background signals were subtracted from the raw LIT images, where the background was calculated from the raw images at |*μ*_0_*H*_*z*_| = 0.15 T and *ε*_*x*_ = 0%. Since the sample has a perpendicular ***M*** under any *H*_*z*_ at *ε*_*x*_ = 0%, the AEE-induced ***J***_q_ is generated perpendicular to the wire direction. In fact, generation and absorption of heat along the edges of the wire is observed as shown in Fig. 4c, which is consistent with the signal of the AEE with the perpendicularly magnetized configuration^7^. The direction of heat generation and absorption is opposite for the cases (A, −0.15 T) and (B, +0.15 T) because of the reversed ***M***. The result for the case (C, 0 T) is almost the same as that for the case (B, +0.15 T) because of the negative remanent ***M***.

With increasing *ε*_*x*_, the perpendicular magnetic anisotropy of the sample is reduced, and ***M*** points toward the in-plane direction at low *H*_*z*_, as shown in Fig. 4a (see the curve for *ε*_*x*_ = 1.2%). In response to this behavior, the amplitude of the AEE-induced Δ*T* at the wire edges for the case (C, 0 T) decreased with *ε*_*x*_, as observed in the images in Fig. 4c–e. Importantly, the Δ*T* signal at the wire edge disappeared at *ε*_*x*_ = 1.2%, indicating that the in-plane ***J***_q_ was almost eliminated. Note that the Δ*T* signal at regions I and V at *ε*_*x*_ = 1.2% is similar to that in the case of the Ni sample. This means that the 90° switching of ***J***_q_ from the in-plane to perpendicular direction is realised as a result of the perpendicular to in-plane (along *x* axis) ***M*** switching due to the tensile strain. Although a patchy pattern in Fig. 4e reflects randomness of the x-component of magnetization, the sign of the AEE signals indicate that a substantial portion of magnetization aligns along the +x direction in the panel C of Fig. 4e, which is possibly due to a small tilt of the magnetic field.

## Discussion

Here, we discuss the origin of the observed Δ*T* signals for the Ni and TbFeCo samples. Since both samples have the Pt adjacent layer, which can generate the spin Hall effect-induced spin current^18^, not only the AEE but also the SPE can be a source of the heat current^5,6^. Because of the different symmetries of the AEE and the SPE, the Δ*T* signals in the TbFeCo sample are purely attributed to the AEE if ***M*** is saturated along the *z* axis, while the SPE contribution is eliminated^7^. In contrast, a contribution from the SPE may exist in the Ni and TbFeCo samples in the in-plane magnetized configuration^7^. Nevertheless, the strain-induced control of the heat current demonstrated here is valid, even in the presence of an SPE contribution. To discuss the magnitude of the current-induced temperature modulation in the TbFeCo sample, it is important to recall that Δ*T* is proportional to the length of the ferromagnetic material along the heat current^5^. Since the width of the TbFeCo sample (200 μm) is much larger than the thickness (6 nm), the Δ*T* signal in the perpendicularly magnetized configuration should be several orders of magnitude greater than that in the in-plane magnetized configuration. However, the difference in the Δ*T* magnitude between these configurations is much smaller than this expectation, as shown in Fig. 4e. The possible explanations for this situation are the following: (i) heat loss from the TbFeCo layer to the substrate and black ink (see Methods) reduces the temperature modulation created in the sample plane^7^, and (ii) the lock-in frequency *f* dependence of the temperature modulation is different between the two configurations^13^, and the magnitude of the AEE signals in the perpendicularly magnetized configuration decreases with increasing *f*, while that in the in-plane magnetized configuration is almost independent of *f* (note that all LIT measurements were performed at the high *f* value of 25 Hz). Judging from the previously reported studies^7,13^, possibility (i) seems to be the dominant reason. Another important point to be discussed is the difference between the Δ*T* signals of the Ni and TbFeCo samples in the in-plane magnetized configurations. The Δ*T* magnitudes at regions I and V for the latter [Fig. 4e,c] are much smaller than those for the former [Fig. 3b,c]. This result may be attributed to the thin magnetic layer (short length along the heat current^5^), the multi-domain state and the small AEE and/or SPE coefficients in the TbFeCo sample. For further efficient strain control of the heat currents, it is important to look for a material with both high AEE and/or SPE coefficients and good magnetoelastic properties.

We also mention the Δ*T* signals appearing around the corners of the Π-shaped wires, *i*.*e*., in regions II and IV. In the TbFeCo sample, Δ*T* clearly appears as the strain is applied. This is consistent with a signal from the anisotropic magneto-Peltier effect (AMPE) when ***M*** aligns along the ±*x* direction and the sign of the AMPE coefficient is opposite to that of bulk Ni^12^, while the sign of the AEE coefficient is the same as Ni. Although the AMPE should also occur in the Ni sample, Δ*T* is negligibly small compared with the AEE [Fig. 3b,c] because the high thermal conductivity of the sapphire substrate reduces the magnitude of the AMPE-induced temperature modulation, while the AEE signal in the in-plane magnetized configuration is almost independent of the thermal conductivity of the substrate^13^.

Our experiment demonstrates that the direction of the heat currents due to the spin-caloritronic and/or magneto-thermoelectric phenomena is controlled by a mechanical strain. This suggests that such heat currents can be controlled even by electrical methods, *e*.*g*., piezoelectrical control of ***M***^19,20^ and electrical gating-induced magnetic easy axis switching^21,22^ or magnetic phase transition^23,24^, though the latter are fundamentally different from the strain-mediated phenomenon. Control of ***M*** using the above methods and the challenges of enhancing Δ*T* may realise a versatile temperature controller or heat current switch, which paves the way for thermal management in increasingly complex electronic and spintronic circuits.

## Methods

### Sample fabrication

For the Ni sample, a 15-nm-thick Ni film with a 3-nm-thick Pt cap layer was deposited on a 100-μm-thick c-plane sapphire substrate by rf sputtering. By covering the substrate with a metal mask during deposition, a 200-μm-wide Π-shaped wire was formed. For the TbFeCo sample, Pt (4 nm)/Tb_21_Fe_67_Co_12_ (6 nm)/Pt (4 nm) layers were deposited on a 50-μm-thick PEN substrate (Q65H, Teijin DuPont) by dc magnetron sputtering. The layers were defined into a Π-shaped wire by photolithography and Ar ion milling.

### Strain machines

A three-point bending machine made of brass was used for applying strain to the Ni sample. The strain applied to the sample surface was calculated using the displacement of the sample centre and the thickness of the substrate. For the experiment using the TbFeCo sample, a screw-driven tensile machine made of brass was used. In this case, the value of the applied strain was calibrated using the elongation of the sample measured via a microscope and the rotation number of the screw.

### Hall measurement

The magnetization curves under the tensile strain in the TbFeCo sample shown in Fig. 4a were measured using a Hall bar-shaped TbFeCo with a 30-μm-wide channel, which was fabricated using photolithography and Ar ion milling. A direct current with a magnitude of 100 μA was used to measure *R*_Hall_.
